# Perinatalna Opieka Paliatywna Realizowana w Oddziale Położniczym i Neonatologicznym We Współpracy z Hospicjum Dla Dzieci – Doświadczenia własne

**DOI:** 10.34763/devperiodmed.20192304.253262

**Published:** 2021-01-29

**Authors:** Agnieszka Jalowska, Joanna Krzeszowiak, Agnieszka Stembalska, Krzysztof Szmyd, Mariusz Zimmer, Gizela Jagielska, Małgorzata Raś, Agnieszka Pasławska, Agnieszka Szafrańska, Dorota Paluszyńska, Tomasz Fuchs, Karolina Pesz, Maria Sąsiadek, Barbara Królak-Olejnik, Robert Śmigiel

**Affiliations:** 1 Katedra i Klinika Neonatologii, Uniwersytet Medyczny we Wrocławiu, Wrocławiu Polska; 2 Katedra Pediatrii, Zakład Propedeutyki Pediatrii i Chorób Rzadkich, Uniwersytet Medyczny we Wrocławiu, Wrocławiu Polska; 3 Katedra Genetyki, Uniwersytet Medyczny we Wrocławiu, Wrocławiu Polska; 4 Stowarzyszenie Hospicjum dla Dzieci Dolnego Śląska „ Formuła Dobra’’, Wrocław, Polska; 5 Katedra i Klinika Ginekologii i Położnictwa, Uniwersytet Medyczny we Wrocławiu, Wrocławiu Polska; 6 Oddział Ginekologii i Położnictwa, Powiatowy Zespół Szpitali w Oleśnicy, Oleśnicy Polska; 7 Oddział Kardiologii Dziecięcej, Wojewódzki Szpital Specjalistyczny we Wrocławiu, Wrocławiu Polska

**Keywords:** perinatalna opieka paliatywna, hospicjum perinatalne, wady letalne, perinatal palliative care, perinatal hospice, lethal defects

## Abstract

Wady letalne prowadzą do wewnątrzmacicznego zgonu płodu lub dziecka bezpośrednio po urodzeniu lub we wczesnym okresie niemowlęcym, bez względu na zastosowane leczenie. W przypadku wad letalnych nie ma możliwości skutecznej pomocy dziecku, mimo postępu mi zeadsytcoysnoyw ania najnowocześniejszej aparatury lub terapii. Rodzice, którzy decydują się na urodzenie dziecka z wadą letalną mogą być objęci perinatalną opieką hospicyjną, która ma charakter kompleksowy. Polega ona na wsparciu ciężarnej w okresie przygotowania do porodu, w czasie porodu i po porodzie oraz na wsparciu jej rodziny oraz obejmuje udzielenie rodzicom pełnej informacji o chorobie ich dziecka. Opieka nad dzieckiem po urodzeniu jest nastawiona na ochronę przed uporczywą terapią i zapewnienie dziecku opieki paliatywnej.

**Cel:**

*Wykazanie znaczenia perinatalnej opieki paliatywnej dla kobiet w ciąży, u których wyniki badań prenatalnych wskazywały na ciężkie zaburzenie rozwojowe u płodu o potencjalnie letalnym rokowaniu oraz przedstawienie schematu postępowania według modelu wewnątrzszpitalnego hospicjum perinatalnego.*

**Materiał i metody:**

*Analizą retrospektywną objęto dokumentację 67 pacjentek skierowanych do Programu RAZEM we Wrocławiu w latach 2014-2018 z powodu nieprawidłowych wyników badań prenatalnych (ultrasonograficznych lub/i genetycznych), które wskazywały na ciężkie zaburzenie rozwojowe u płodu o potencjalnie letalnym rokowaniu. Dokonanoanalizy danych socjodemograficznych, danych klinicznych rozpoznania choroby u płodu, przebiegu ciąży i porodu, trybu postępowania w okresie prenatalnym, podczas porodu i po urodzeniu się dziecka.*

**Wyniki:**

*Do Programu zostało skierowanych 67 kobiet w wieku 20–43 lat (średnio 31,2), które zgłaszały się w okresie od 15 do 39 tygodnia ciąży (średnio w 25. tygodniu ciąży). Do opieki paliatywnej zakwalifikowano 57 kobiet, czyli 85% skierowanych do programu. Opiekę paliatywną kontynuowano u 51 pacjentek, ponieważ 6 kobiet w trakcie procesu diagnostycznego zdecydowało się na zakończenie ciąży (10,5%). Najczęstszymi zaburzeniami u płodów były aberracje chromosomowe, wady OUN i wady nerek. W 95% przypadków doszło do obumarcia wewnątrzmacicznego płodu lub śmierci noworodka.*

**Wnioski:**

*Perinatalna opieka paliatywna jest niezbędną formą opieki dla kobiet w ciąży, u których wyniki badań prenatalnych wskazują na ciężkie zaburzenie rozwojowe u płodu o potencjalnie letalnym rokowaniu. Model wewnątrzszpitalny hospicjum perinatalnego jest korzystną formą opieki, zapewnia jej spójność i dobrą komunikację w zespole, co wpływa na dobrą jakość opieki.*

## Abstract (en)

Lethal defects lead to the intrauterine death of the fetus or the passing away of the child immediately after birth or in early infancy, regardless of the treatment used. In the case of lethal defects, it is not possible to effectively help the child, despite using the most modern equipment or medicines in the treatment or the progress made by medicine. Parents, who decide to continue the pregnancy, although the fetus has a lethal defect that cannot be cured, may be covered by perinatal hospice care, which is comprehensive and consists in supporting the pregnant woman during the prenatal time, during delivery and after delivery and support of her family, giving full information to the parents about their child's illness. Childcare after birth is focused on protecting the infant from persistent therapy and providing him with appropriate conditions.

**Aim:**

*To demonstrate the role of perinatal palliative care for pregnant women in whom the results of prenatal tests pointed to a severe developmental disorder in the fetus with a potentially lethal prognosis, and to present a pattern of behavior for their hospitalization in the perinatal hospice.*

**Materials and methods:**

*The retrospective analysis included documentation of 67 patients referred to the RAZEM (TOGETHER) Program in Wrocław in 2014-2018 due to abnormal results of (ultrasound and / or genetic) prenatal tests, which indicated a serious developmental disorder in the fetus with potentially lethal prognosis. Analysis was conducted of sociodemographic data, clinical data on fetal diagnosis, pregnancy and delivery, the procedure for prenatal delivery and postnatal birth.*

**Results:**

*67 women aged 20-43 years (mean 31.2) were referred to the RAZEM Program. Out of these, 57 women were enrolled for palliative care, which accounted for 85% of those referred to the program. Palliative care was continued in 51 patients, because 6 women decided to terminate their pregnancy during the diagnostic process (10.5%). The most common abnormalities in the fetuses were chromosomal aberrations, CNS defects and kidney defects. In 95% of the cases, intrauterine fetal death or neonatal death occurred.*

**Conclusions:**

*Perinatal palliative care is an indispensable form of care for pregnant women in whom the results of prenatal tests indicate a serious developmental disorder in the fetus with potentially lethal prognosis. The in-hospital model of a perinatal hospice is a beneficial form of care, as it ensures consistency and good communication in the team, which favourably affects its quality.*

## Wstęp

Wrodzona wada rozwojowa to każda nieprawidłowość anatomiczna powstała w okresie życia płodowego i obecna u dziecka po urodzeniu. Wady wrodzone stwierdza się u około 2-3% żywo i u ok. 10% martwo urodzonych dzieci. Ocenia się, że ponad 50% płodów z ciężkimi wadami rozwojowymi umiera wewnątrzmacicznie [1].

Etiologia wrodzonych wad rozwojowych może być zarówno genetyczna, jak i środowiskowa. W praktyce klinicznej wyróżnia się wady małe i duże. Pierwsze nie pociągają za sobą trwałych skutków medycznych lub/i psychologicznych. Drugie powodują poważne następstwa dla zdrowia płodu lub noworodka [2]. Niektóre wady tej grupy mają charakter letalny, czyli stanowią ciężkie zaburzenia rozwojowe, dla których rokowanie dotyczące przeżycia jest niepewne lub złe. Wady letalne prowadzą do zgonu wewnątrzmacicznego płodu (poronienia/przedwczesnego urodzenia martwego dziecka) lub śmierci dziecka bezpośrednio po urodzeniu lub we wczesnym okresie niemowlęcym bez względu na zastosowane leczenie [2, 3]. W przypadku wad letalnych nie ma możliwości skutecznej pomocy dziecku, mimo postępu medycyny czy zastosowania w leczeniu najnowocześniejszej aparatury lub leków – według aktualnego stanu wiedzy brak jest możliwości terapii przyczynowej [3].

Rozpoznanie wady wrodzonej u płodu, szczególnie wady określanej jako letalna jest trudnym przeżyciem dla rodziców. Z jednej strony muszą zmierzyć się ze „stratą” zdrowego dziecka, na które czekali, z drugiej strony muszą podjąć decyzję dotyczącą dalszego postępowania w ciąży [4]. Zgodnie z prawem polskim w takich przypadkach istnieje możliwość podjęcia decyzji o kontynuacji ciąży łub jej zakończeniu (Ustawa z dnia 7 stycznia 1993 r. o planowaniu rodziny, ochronie płodu ludzkiego i warunkach dopuszczalności przerywania ciąży Dz. U. z 1993 r. Nr 17, poz. 78). Rodzice, którzy decydują się na urodzenie dziecka z wadą letalną, ciężką, niemożliwą do wyleczenia mogą być objęci perinatalną opieką hospicyjną [5]. Jest to kompleksowa opieka obejmująca w pierwszej kolejności wsparcie ciężarnej i jej rodziny oraz udzielenie pełnej informacji rodzicom o chorobie ich dziecka [6]. Działalność hospicjum perinatalnego obejmuje nadzór nad kobietą i dzieckiem w okresie przygotowania do porodu, w czasie porodu i po porodzie [5, 6, 7]. Opieka po urodzeniu jest nastawiona na ochronę przed uporczywą terapią oraz obejmuje wszystkie elementy opieki paliatywnej: medyczny, psychologiczny, duchowy i socjalny zarówno nad dzieckiem, jak i jego rodziną [8].

W zespole tworzącym hospicjum perinatalne znajdują się nie tylko lekarze, ale i położne, pielęgniarki, psycholodzy i osoby duchowne.

Aktywność medyczna w ramach hospicjum perinatalnego skupia się przede wszystkim na szczegółowym informowaniu o możliwych procedurach medycznych i ich konsekwencjach dla matki i dziecka. Bez takiego przygotowania podejmowanie decyzji po porodzie w trybie pilnym staje się źródłem dodatkowego obciążenia, rzutującego na funkcjonowanie całej rodziny, a w dalszej kolejności, przeżywanie procesu żałoby. Innym aspektem związanym z medyczną częścią działalności hospicjum perinatalnego jest tworzenie ośrodków medycznych, w których rodzice dziecka z wadą letalną mogą otrzymać odpowiednią pomoc. Hospicjum perinatalne jest także przygotowaniem do opieki hospicyjnej w warunkach domowych nad nieuleczalnie chorym dzieckiem po urodzeniu. Dotyczy to noworodków w stanie na tyle stabilnym, które mogą być wypisane ze szpitala pod domową opiekę rodziców. Opieka hospicyjną jest prowadzona również w momencie śmierci dziecka, a nawet przedłuża się na czas po jego śmierci i obejmuje okres żałoby rodziców.

W procesie decyzyjnym dotyczącym dalszego postępowania po postawieniu diagnozy poważnej wady rozwojowej płodu, uczestniczy wielu specjalistów, w tym: lekarze położnicy prowadzący ciążę i diagnostykę prenatalną, specjaliści genetyki klinicznej poszukujący przyczyny wad rozwojowych, kardiolodzy i chirurdzy dziecięcy konsultujący wady strukturalne u płodu, a także psycholodzy i w razie potrzeby osoby duchowne. Zadaniem tych osób jest postawienie właściwego rozpoznania, udzielenie rzetelnej informacji na temat choroby płodu/dziecka, ustalenie zasad postępowania, w tym rokowania oraz udzielenie wsparcia psychologicznego matce dziecka i jej rodzinie [9, 10]. W działania hospicjum perinatalnego włączeni są również lekarze neonatolodzy i medycyny paliatywnej, którzy są zaangażowani w opiekę nad dzieckiem już po jego urodzeniu. Nieoceniona jest w tym procesie rola personelu pielęgniarskiego i położnych.

Miarą sukcesu hospicjum perinatalnego jest stworzenie odpowiedniego zespołu wielu specjalistów, często pracujących w różnych ośrodkach, których działanie koordynuje jedna osoba pozostająca w stałym kontakcie z rodziną. Niezmiernie ważnym aspektem współdziałania zespołu hospicjum perinatalnego jest dobra komunikacja między osobami decyzyjnymi oraz między personelem medycznym a rodzicami [11]. Dzięki temu wszechstronną opieką zostaje otoczony płód i dziecko po urodzeniu (przez położnika-ginekologa, genetyka, neonatologa, położną czy pielęgniarkę), matka (przez ginekologa, położną, psychologa czy dodatkowo osobę duchowną), a także ojciec i cała rodzina, w tym rodzeństwo pacjenta hospicyjnego (przez psychologa czy dodatkowo osobę duchowną).

Podsumowując, do zadań perinatalnej opieki paliatywnej należy m.in.:

– udzielenie rodzicom rzeczowej informacji na temat choroby płodu/noworodka;
– stworzenie interdyscyplinarnego planu postępowania, uwzględniającego różne scenariusze tego, co może się wydarzyć podczas ciąży, porodu i życia dziecka po urodzeniu; planowanie z rodziną i przygotowywanie jej na trudne doświadczenia [12];
– opieka medyczna nad dzieckiem i ochrona przed uporczywą terapią [7, 8];– wsparcie psychologiczne, socjalne i w razie potrzeby duchowe dla rodziny, w trakcie trwania ciąży, w okresie okołoporodowym, a także opieka w czasie żałoby.

W Polsce na przestrzeni ostatnich lat powstało kilkanaście hospicjów perinatalnych [13]. Od 2018 konsultacje rodzin skierowanych do takiej opieki są refundowane przez NFZ. (Zarządzenie Prezesa Narodowego Funduszu

Zdrowia z dnia 31 lipca 2018 r. w sprawie określenia warunków zawierania i realizacji umów w rodzaju opieka paliatywna i hospicyjna).

W 2014 roku we Wrocławiu zaczęło funkcjonować hospicjum perinatalne w ramach Programu RAZEM. Było to wspólne przedsięwzięcie wrocławskich placówek: Uniwersyteckiego Szpitala Klinicznego, Poradni Genetycznej Fundacji Uniwersytetu Medycznego (FUM) oraz Hospicjum dla Dzieci Dolnego Śląska i Fundacji Evangelium Vitae. Program jest skierowany do kobiet w ciąży, u których wyniki badań prenatalnych (ultrasonograficznych lub/ i genetycznych) wskazywały na ciężkie zaburzenie rozwojowe u płodu o potencjalnie letalnym rokowaniu. Nazwa Programu „RAZEM” odzwierciedlała potrzebę dobrej komunikacji między zaangażowanymi specjalistami dla wspólnego celu jakim jest realizacja perinatalnej opieki hospicyjnej. W tym celu wypracowano model opieki perinatalnej wewnątrzszpitalny, który wydawał się najbardziej korzystny z uwagi na skupienie kluczowych działań hospicyjnych podczas porodu dziecka (martwo lub żywo urodzonego) i bezpośrednio po jego urodzeniu się. Opieka nad rodziną była prowadzona przez koordynatorów pracujących w szpitalu, jednak do Programu pacjentki były kierowane przez lekarzy genetyków klinicznych lub ginekologów-położników, do których rozesłano informację na temat założeń i celów Programu RAZEM.

## Cel pracy

Celem pracy jest wykazanie znaczenia perinatalnej opieki paliatywnej dla kobiet w ciąży, u których wyniki badań prenatalnych (ultrasonograficznych lub/i genetycznych) wskazywały na ciężkie zaburzenie rozwojowe u płodu o potencjalnie letalnym rokowaniu oraz przedstawienie schematu postępowania według modelu wewnątrzszpitalnego hospicjum perinatalnego.

## Materiał i metody

Dokonano analizy retrospektywnej danych z dokumentacji 67 pacjentek, skierowanych do Programu RAZEM we Wrocławiu w latach 2014-2018 z powodu nieprawidłowych wyników badań prenatalnych (ultrasonograficznych lub/i genetycznych), które wskazywały na ciężkie zaburzenie rozwojowe u płodu o potencjalnie letalnym rokowaniu. Ocenione zostały informacje socjodemograficzne, dane kliniczne dotyczące rozpoznania choroby u płodu, przebieg ciąży i porodu, tryb postępowania w okresie prenatalnym porodu i po urodzeniu się dziecka.

W ramach Programu koordynator perinatalnej opieki paliatywnej organizował pacjentce i ojcu dziecka (rodzinie) spotkania z położną i neonatologiem, pilotując rodzinę przez cały czas trwania ciąży, podczas porodu oraz po urodzeniu się dziecka i po jego śmierci. Ponadto rodzina była kierowana na konsultacje do ginekologów-położników i kardiologa dziecięcego, a później, w zależności od wskazań, także na konsultację do genetyka klinicznego, chirurga dziecięcego, neurologa oraz do lekarzy innych specjalności. Konsultacje specjalistyczne miały na celu ocenę stanu płodu, ustalenie diagnozy i prawdopodobnego rokowania dla płodu i dziecka (zgon w okresie prenatalnym lub okołoporodowym lub opieka długoterminowa w oddziale noworodkowym i poza nim w warunkach domowych) oraz wydanie wspólnej opinii na temat dalszego postępowania, z uwzględnieniem postępowania okołoporodowego. Każda rodzina otrzymywała kartę konsultacyjną Programu opisującą propozycję postępowania położniczego i okołoporodowego, postępowania z noworodkiem po urodzeniu. Ponadto każdej rodzinie proponowano opiekę psychologa i położnej (indywidualna szkoła rodzenia). Większość spotkań odbywało się na terenie Uniwersyteckiego Szpitala Klinicznego.

Poszczególne wizyty obejmowały konsultacje specjalistyczne, podczas których omawiano:

- w trakcie konsultacji ginekologiczno-położniczej:
  - dalszy przebieg ciąży i opiekę położniczą,
  - monitorowanie porodu,
  - akceptacja wewnątrzmacicznej śmierci płodu podczas porodu,
  - preferowany sposób porodu siłami natury oraz ustalenie wskazań do ewentualnego cięcia cesarskiego,
  - zorganizowanie sali porodów rodzinnych i intymnych warunków porodu;
- w trakcie konsultacji neonatologicznej:
  - odstąpienie od czynności i zabiegów resuscytacyjnych,
  - możliwość pozostawienia dziecka z matką w kontakcie „skóra do skóry” oraz przebywanie z ojcem,
  - zabezpieczenie warunków cieplnych przez kontakt „ skóra do skóry”, owinięcie w pieluszki,
  - zapewnienie możliwego w danej sytuacji żywienia,
  - zapewnienie środków łagodzących objawy, w tym ból,
  - możliwość obrzędów religijnych u dziecka np. chrzest,
  - utrwalenie odcisku stopki lub dłoni,
  - omówienie postępowania po śmierci dziecka.

Karty konsultacyjne były podpisywane przez rodziców i specjalistów z danej dziedziny. Ustalone w nich postępowanie było wiążące dla zespołu medycznego Uniwersyteckiego Szpitala Klinicznego, co miało na celu przygotowanie się zespołów medycznych do proponowanego postępowania oraz uniknięcie powtarzania ustaleń okołoporodowych w momencie porodu. Karty otrzymywali zarówno rodzice, jak i lekarze bloku porodowego Kliniki Położnictwa i Ginekologii oraz lekarze Kliniki Neonatologii. Informacje zawarte w kartach stanowiły rodzaj planu porodowego, którego tworzenie jest jednym z zadań perinatalnej opieki paliatywnej.

## Wyniki

Do Programu zostało skierowanych 67 kobiet w wieku 20-43 lat (średnia wieku 31,2 lat) między 15 a 39 tygodniem ciąży (średnio w 25. tygodniu ciąży) (Tabela I).

Po wstępnej analizie sytuacji klinicznej płodu (rozpoznanie ultrasonograficzne, genetyczne) do postępowania paliatywnego zakwalifikowano 57 rodzin, czyli 85% skierowanych do Programu. Pozostałym kobietom (10 osób) udzielono wsparcia psychologicznego i opieki wielospecjalistycznej, a także wsparcia ze strony innych rodziców dzieci z daną wadą rozwojową. W połowie tych przypadków rozpoznano prenatalnie trisomię chromosomu 21, a u pozostałych wadę OUN (4 płody) i wadę nerek (1 płód).

**Table I:** Qualification for perinatal care and decisions of patients regarding continuation or termination of pregnancy. Tabela I. Kwalifikacja do opieki i decyzje pacjentek odnośnie kontynuacja lub zakończenia ciąży.

Podział pacjentek na grupy Groups of patient	n	Decyzja matki o zakończeniu ciąży *Terminationof pregnancy*	Decyzjao kontynuacji ciąży *Continuationof pregnancy*
Zakwalifikowane do perinatalnejopieki paliatywnej (POP) *Qualified to perinatal palliative care (POP)*	47 (70,1%)	5	42
Zakwalifikowane do równoległegopostępowania paliatywnego (RPP) *Qualified to parallel palliative care (RPP)*	10 ( 14,9%)	1	9
Niezakwalifikowane do POP ani RPP *Non- qualified to POP and RPP*	10 ( 14,9%)	0	10
**ŁĄCZNI** ***Total***	**67**	**6 (10,5%)**	**61**

Wśród 57 pacjentek zakwalifikowanych do postępowania paliatywnego wyróżniono dwie grupy. Pacjentkom z pierwszej grupy zaproponowano pełną perinatalną opiekę paliatywną (POP) - 47 rodzin (70,7% zgłoszonych do Programu). Z POP skorzystały 42 rodziny. Pięć pacjentek w tej grupy podjęło decyzję o zakończeniu ciąży (Tabela I).

Drugą grupę stanowiło 10 kobiet, u których płodów postawiono prenatalne rozpoznanie choroby, która nie spełniała kryteriów wady letalnej, jednak dodatkowe okoliczności (dodatkowe wady narządów wewnętrznych, przebieg ciąży) stwarzały ryzyko przedwczesnej śmierci płodu/noworodka. W tej grupie u 9 pacjentek, obok standardowej opieki medycznej, zastosowano niektóre elementy opieki paliatywnej (określone jako tzw. **równoległe postępowanie paliatywne),** takie jak indywidualne wsparcie psychologiczne i duchowe rodziców, omawianie możliwych scenariuszy przebiegu ciąży, porodu i okresu noworodkowego, kierowanie do poszczególnych specjalistów w zależności od potrzeb i wskazań medycznych, proponowanie i organizowanie wsparcia ze strony rodzin, które mają dzieci z podobnymi schorzeniami, a także wsparcie po śmierci dziecka. W tej grupie jedna pacjentka podjęła decyzję o zakończeniu ciąży.

Łącznie wśród wszystkich kobiet, zakwalifikowanych do jakiejkolwiek formy postępowania paliatywnego (57 osób) – 6 (10,5%) podjęło decyzję o zakończeniu ciąży. Te kobiety/rodziny mogły w ramach programu skorzystać z opieki psychologicznej.

W grupie pacjentów zakwalifikowanych do pełnej perinatalnej opieki paliatywnej (42 przypadki) zdiagnozowano przede wszystkim aberracje chromosomowe (18 przypadków, 42,9%), z czego najczęściej występowała trisomia chromosomu 18 (Tabela II). Do pozostałych dominujących w tej grupie schorzeń należały wady ośrodkowego układu nerwowego (8 przypadkach, 19%, najczęściej bezmózgowie, ale także masywne wodogłowie i wodomózgowie) oraz ciężkie wady nerek (6 przypadków 14,3%, agenezja nerek, obustronna wielotorbielowatość nerek, masywna uropatia zaporowa).

**Table II:** Group of patients receiving Perinatal Palliative Care (Legend: HD – home hospice, PSN – natural delivery, CC – caesarean section). Tabela II. Grupa pacjentów objętych Perinatalną Opieką Paliatywną ( Legenda: HD – hospicjum domowe, PSN – poród siłami natury, CC – cięcie cesarskie).

Rozpoznanie u płodu *Diagnosis*		Sposób zakończeniaciąży *Delivery*		Zgon Death			HD
	n	PSN	CC	Prenatalnie *prenatal*	<24 godz *<24 hours*	>24 godz *>24 hours*	
Aberracje chromosomowei choroby monogenowe *Chromosomal aberrationand single gene defects*	18 (42,9%)	16	2	10	5	3	2
Trisomia 18 *Trisomy 18*	7	6	1	4	1	2	1
Trisomia 13 *Trisomy 13*	4	3	1	1	1	1	1
Triploidia *Triploidy*	3	3	-	3	-	-	-
Trisomia 22 *Trisomy 22*	1	1	-	1	-	-	-
Z. Smitha-Lemliego-Opitza(SLOS) *Smith-Lemli-Opitz syndrome(SLOS)*	1	1	-	-	1	-	-
Dysplazja tanatoforyczna *Thanatophoric dysplasia*	1	1	-	-	1	-	-
Złożona aberracja chromosomowaz wielowadziem *Complex chromosomalaberration with multidefects*	1	1	-	-	1	-	-
**Wady OUN** ***CNS defects***	**8 (19%)**	**5**	**3**	**2**	**5**	**1**	-
Bezmózgowie *Anencephaly*	6	4	2	1	5	-	-
Wodomózgowie *Hydroanencephaly*	1	-	1	-	-	1	-
Masywne wodogłowiei przepuklina oponowo-rdzeniowa *Severe hydrocephalus and spina bifida*	1	1	-	1	-	-	-
**Wady nerek** ***Kidney defects***	**6 (16,3%)**	4	2	1	5	-	-
Agenezja nerek *Renal agenesis*	3	3	-	1	-	-	-
Uropatia zaporowa *Ureter obstructive defects*	2	-	2	-	2	-	-
Wielotorbielowatość nerek *Bilateral polycystic kidney disease*	1	1	-	-	1	-	-
**Zespoły z wielowadziem,bez określonej przyczyny** ***Multidefects syndromewithout known ethiology***	**9 (21,4%)**	**8**	**1**	**3**	**5**	-	-
**Obrzęk uogólniony** ***Hydrops fetalis***	1	1	-	1	-	-	-
**ŁĄCZNIE** ***TOTAL***	**42**	**34**	**8**	**16**	**20**	**4**	**2**
%				95%			

Analizując przebieg ciąży pacjentek zakwalifikowanych do pełnej paliatywnej opieki, w 17 przypadkach na 42 (40%) doszło do wewnątrzmacicznego obumarcia płodu, natomiast w 23 przypadkach dzieci urodziły się żywe, ale zmarły w Szpitalu. Dwadzieścia jeden dzieci zmarło na bloku porodowym w 1. dobie życia (najwięcej w pierwszych 2. godzinach życia), natomiast dwoje dzieci zmarło w oddziale neonatologicznym (jedno w 1. a drugie w 2. tygodniu życia). Łącznie w tej grupie w okresie prenatalnym i okołoporodowym, w szpitalu zmarło 95% dzieci. Tylko dwoje pacjentów zostało wypisanych do domu pod opiekę hospicjum domowego dla dzieci. Jedno dziecko, z rozpoznaniem trisomii chromosomu 13, zmarło w drugim miesiącu życia, a drugie, z rozpoznaniem trisomii chromosomu 18, zmarło w trzecim miesiącu. Wszystkie matki objęte perinatalną opieką paliatywną chciały zobaczyć i przytulić dziecko. Wszystkie rodziny wyraziły chęć zabezpieczenia pamiątek po dziecku (kocyk, odcisk stopy czy opaska) i wielu z nich wysyłało do członków zespołu Programu RAZEM informację o dużym znaczeniu tych pamiątek.

W grupie tzw. równoległego postępowania paliatywnego (9 pacjentek) oprócz trisomii 21, która występowała najczęściej (3 przypadki), diagnozowano również wady ośrodkowego układu nerwowego (2 przypadki, masywne wodogłowie nadnamiotowe, holoprozencefalię semilobarną), zespół genetyczny (2 przypadki, zespół Smitha-Lemliego-Opitza oraz hipofosfatazja), a także wielowadzie o nieokreślonej etiologii (2 przypadki).

W tej grupie, w jednym przypadku doszło do wewnątrz­macicznego obumarcia płodu (rozpoznanie: trisomia 21 i obrzęk płodu); jedno dziecko zmarło w oddziale intensywnej terapii (rozpoznanie: trisomia 21 z zarośnięciem dwunastnicy i wspólnym kanałem przedsionkowokomorowym), dwoje dzieci zmarło po wypisaniu do domu (jedno z powodu wady serca w przebiegu trisomii 21 w 6. miesiącu życia, drugie z powodu wielowadzia w 2. roku życia. Pięcioro dzieci z tej grupy żyje. Jedno dziecko z powodu ciężkiej hypofosfatazji nadal przebywa w szpitalu; a pozostałych czworo (w wieku od 8 miesięcy do 2 lat) przebywa w domu. Troje z tych dzieci jest pod opieką Hospicjum Domowego dla Dzieci.

**Table III:** *The group of patients covered by the so-called parallel palliative care (Legend: HD* - *home hospice, PSN* - *natural delivery, CC - caesarean section*). Tabela III. Grupa pacjentόw objętych tzw. rόwnolegtym postępowaniem paliatywnym ( Legenda: HD - hospicjum domowe, PSN - porod sitami natury, CC- cięcie cesarskie).

Rozpoznanie u płodu Diagnosis		Sposób zakończenia ciązy Delivery		Losy dziecka Follow-up					
				Zgon Death				Przeżycie > 2 rż. Alive > 2 y	
	n	PSN	CC	Prenatalnie Prenatal	<24 godzin <24 hours	< 1 mies. życia <1 month	1-2 rż 1-2 years	Bez opieki HD Without HD	HD
Trisomia 21 z wadami narządowymi *Trisomy 21 with organ defects*	**3**	**1**	**2**	**1**	**-**	**1**	**1**	**-**	**-**
Wielowadzie *Multidefects syndrome*	**2**	**-**	**2**	**-**	**-**	**-**	**1**	**1**	**-**
Hipofosfatazja *Hypophosphatasia*	**1**	**-**	**1**	**-**	**-**	**-**		**-**	**1**
Hotoprosencefalia semiobarna. *Holoprosencephaly* *semilobar*	**1**	**-**	**1**	**-**	**-**	**-**		**1**	**-**
Zespół Smitha, Lemliego i Opitza *Smith-Lemli-Opitz* *syndrome*	**1**	**1**	**-**	**-**	**-**	**-**		**-**	**1**
Masywne wodogłowie *Severe hydrocephalus*	**1**	**-**	**1**	**-**	**-**	**-**		**-**	**1**
**ŁĄCZNIE** ***Total***	**9**	**2**	**7**	**1**	**-**	**1**	**2**	**2**	**3**

W grupie pacjentek objętych perinatalną opieką paliatywną porody odbywały się najczęściej przedwcześnie (78,9%). Były to głównie porody siłami natury (34 pacjentki), znacznie rzadziej, bo tylko w 8. przypadkach przez cięcie cesarskie. Do zabiegu cięcia cesarskiego kwalifikowano najczęściej ze wskazań matczynych (5 przypadków), w 1. przypadku z woli rodziców, a w 2. pozostałych sytuacjach z powodu budowy anatomicznej dziecka.

## Dyskusja

Wśród 57 kobiet, u których płodów stwierdzono ciężkie wady rozwojowe, w tym letalne i zostały skierowane do Programu RAZEM, 51 zdecydowało się na objęcie opieką przez hospicjum perinatalne. Tylko 6 kobiet (10,5 %) zdecydowało się na zakończenie ciąży. Żadna z pozostałych osób nie zrezygnowała z proponowanej opieki paliatywnej. Na podstawie zgromadzonych danych można zatem stwierdzić, że perinatalna opieka paliatywna jest alternatywą dla kobiet, które chcą kontynuować ciążę, mimo rozpoznania ciężkiej choroby u płodu [4, 7, 8, 13].

W grupie pacjentów objętych pełną perinatalną opieką paliatywną (POP) wykazano, że w większości przypadków stwierdzonych ciężkich wad płodu dochodzi do obumarcia wewnątrzmacicznego lub zgonu okołoporodowego. Łącznie 95% zgonów płodów i noworodków odbywa się zatem w oddziale ginekologiczno-położniczym na sali porodowej lub w oddziale noworodkowym. Umiejscowienie hospicjum perinatalnego w szpitalu wpływa więc pozytywnie na kompleksowość, ciągłość i spójność opieki [14, 15].

Dane uzyskane od pacjentek, u których zastosowano tzw. równoległe postępowanie paliatywne (RPP, kobiety w ciąży, u których postawiono prenatalne rozpoznanie choroby płodu, która nie spełniała w momencie rozpoznania kryteriów wady letalnej, jednak dodatkowe okoliczności tj. dodatkowe wady narządów wewnętrznych, przebieg ciąży, stwarzały ryzyko przedwczesnej śmierci płodu/noworodka), sugerują włączenie ich do perinatalnej opieki paliatywnej z zaznaczeniem pewnych odrębności w rokowaniu i dalszym postępowaniu. Taka forma opieki spełnia założenia ewoluującego paradygmatu medycyny paliatywnej. W jego założeniach opieka paliatywna nie tylko następuje po opiece aktywnej (czyli standardowym prowadzaniu ciąży, porodu oraz pełnym leczeniu przyczynowym u noworodka) ale jej elementy są wprowadzane w sposób równoległy w trakcie postępowania diagnostyczna - leczniczego i przechodzą płynnie do opieki wyłącznie paliatywnej [15].

Decyzje podejmowane przez specjalistów zaangażowanych w jakąkolwiek formę perinatalnej opieki paliatywnej, odnoszące się do rodzaju i zakresu postępowania, mogą się zmieniać i płynnie przechodzić między aktywnym leczeniem i opieką paliatywną. Planowanie opieki staje się procesem dynamicznym i jest nieustannie weryfikowane, ale przez cały czas dąży do zapewnienia jak największego komfortu dziecku i jego rodzinie.

Ważnym aspektem opieki paliatywnej, która jest proponowana w ramach programu Razem, to spójność opieki i komunikacja w zespole, o czym świadczy fakt wiernej realizacji ustalonego wcześniej planu postępowania zarówno na bloku porodowym, jak i oddziale neonatologicznym. Co więcej, postępowanie było w pełni akceptowane przez rodziców i personel szpitala. O spójności i dobrej komunikacji może świadczyć także mały odsetek cięć cesarskich, które u pacjentek zakwalifikowanych do opieki paliatywnej były wykonywane głównie ze wskazań ginekologicznych.

Skoncentrowanie pracy hospicjum perinatalnego w oddziałach szpitalnych (ginekologiczno-położniczym i neonatologicznym), a także wczesne objęcie opieką i tworzenie kart konsultacyjnych (zawierających plan postępowania) umożliwiało wsparcie w sytuacji wczesnych strat ciąży (obumarcia wewnątrzmaciczne), które stanowiły 40% przypadków w grupie objętej pełną opieką paliatywną (POP). Wyniki te dowodzą po raz kolejny, że opieka paliatywna może i powinna być z powodzeniem wprowadzana w zakres działalności każdego szpitala ginekologiczno–położniczego [14, 16].

Realizowanie zadań i idei hospicjum perinatalnego (model wewnątrzszpitalny hospicjum) wpływa też na zmianę postaw, poglądów personelu oddziału położniczego i neonatologicznego [16]. Dotyczy to nie tylko lekarzy, ale i położnych/pielęgniarek. Zauważono, że personel dostrzega zalety stosowania elementów opieki paliatywnej nad płodami i noworodkami [17, 18]. Wobec braku możliwości aktywnego leczenia, położne/ pielęgniarki często twierdziły, że nie mają nic do zaoferowania kobiecie w ciąży/noworodkowi/rodzinie. Wprowadzenie praktycznych aspektów POP, takich jak opieka medyczna (zapewnienie komfortu cieplnego, leków p/bólowych, itp.), towarzyszenie, tworzenie wspomnień, dały personelowi konkretne narzędzie do pomocy swoim podopiecznym. Z obserwacji wynika, że zmiana mentalności pozwoliła również na zwiększenie poczucia sensu takiej formy opieki, podkreślając np. niezastąpioną rolę położnych [19].

Wśród płodów objętych opieką paliatywną najczęściej rozpoznawano aberracje chromosomowe. Wśród nich najczęstszą była trisomia chromosomu 18. Dane statystyczne Orphanet wskazują, że ok 50% urodzonych żywo noworodków z trisomią chromosomu 18 przeżywa dłużej niż tydzień [9, 20]. Z przedstawionych w pracy przypadkach, tylko jedno z siedmiorga dzieci żyło dłużej niż kila godzin i zostało wypisane do domu, a ponad połowa (4/7) obumarła wewnątrzmaciczne. Biorąc pod uwagę żywe urodzenia dzieci z trisomią 18, w naszych badaniach dłużej niż tydzień przeżyło 1/3 dzieci. Podkreślić również należy, że do domu zostały wypisane właśniedzieci z aberracjami chromosomowymi, co może sugerować potrzebę najściślejszej współpracy z Hospicjami Dziecięcymi właśnie w grupie takich pacjentów.

Przedstawiona praca nie obejmowała oceny jakości zaproponowanej perinatalnej opieki paliatywnej, w szczególności oceny takiej formy opieki przez rodziców. Z dotychczasowych badań prowadzonych miedzy innymi przez Wool i wsp. wynika, że perinatalną opiekę paliatywną pozytywnie ocenia ponad 80% rodzin [21]. W zgromadzonym materiale własnym, znalazło się wiele spontanicznych, ale przekazanych w formie pisemnej opinii rodziców, objętych opieką zespołu hospicjum perinatalnego. Oto niektóre z nich:

*„Dziękujemy osobie która od samego początku zaopiekowała się nami, skierowała do odpowiednich osób i pomogła przejść całą ciężką ścieżkę dając wsparcie i poczucie, że nie jesteśmy sami. Opieka, spokój oraz zaakceptowanie podjętej przez nas decyzji pozwoliło nam na duży komfort psychiczny oraz pewność, że my i nasze dziecko jesteśmy w dobrych rękach.*

*Dziękujemy także paniom położnym za przekazanie fachowej wiedzy dotyczącej porodu, która pozwoliła mi przygotować się ze spokojem na narodziny dziecka.*

*(...) Dziękujemy za atmosferę jaką Panie stworzyły podczas porodu. Dzięki doświadczeniu pań położnych poród przebiegł w komfortowych dla mnie warunkach bez większego bólu, wysiłku i co najważniejsze bez jakiegokolwiek urazu psychicznego. (...) Dzięki Wam my oraz nasze dziecko mogło przebywać w bardzo dobrych warunkach, pod profesjonalną opieką i bez niepotrzebnego stresu” Rodzice Gabrysia*

*(...) czas z Jurkiem po porodzie pozostanie w nas na zawsze! Czas tak intymny, przeznaczony tylko nam! Wszystko się szybko toczy i naprawdę pozostają tylko zdjęcia - bardzo za nie dziękuję, i kilka najdroższych sercu pamiątek, jak odcisk stopek, czapeczka, kocyk...! bez Państwa chyba nawet nie pomyślelibyśmy o tym, a po czasie dopiero dochodzi się do przekonania jakie to jest ważne!” Rodzice Jurka*

*„Chcieliśmy podziękować za okazane zainteresowanie i wsparcie; dało się to odczuć, że Julia była traktowana wyjątkowo i z wielkim uczuciem.” Rodzice Julki*

## Podsumowanie

Perinatalna opieka paliatywna jest niezbędną formą opieki dla kobiet w ciąży, u których wyniki badań prenatalnych (ultrasonograficznych lub/i genetycznych) wskazują na ciężkie zaburzenie rozwojowe u płodu o potencjalnie letalnym rokowaniu.

Model wewnątrzszpitalny hospicjum perinatalnego jest korzystną formą opieki, zapewnia spójność działania i dobrą komunikację w zespole. Wpływa to na dobrą jakość opieki, biorąc pod uwagę fakt, że 95% zakwalifikowanych do opieki paliatywnej płodów umiera wewnątrzmacicznie lub w okresie okołoporodowym (najczęściej na bloku porodowym pod opieką położnych i neonatologów).

Współpraca interdyscyplinarnego zespołu pozwala w sposób ciągły, płynny i spójny prowadzić opiekę zarówno w szpitalu, poradniach specjalistycznych, jak i domu pacjenta. Jest ona oparta na trosce o rodzinę, która przeżywa stratę dziecka.

Istotne wydaje się wprowadzenie elementów perinatalnej opieki paliatywnej, tzw. równoległego postepowania paliatywnego w sytuacjach ciężkich schorzeń wrodzonych płodu o niejednoznacznym rokowaniu. W części przypadków zakres wad rozwojowych u płodu zwiększa ryzyko jego wewnątrzmacicznego obumarcia.

Ponadto po urodzeniu się chorego dziecka z wadami rozwojowymi mogą pojawić się nowe obciążenia wynikające np. z porodu przedwczesnego czy inne powikłania, co zwiększa ryzyko przedwczesnej śmierci dziecka.
